# Macroscale fluorescence imaging against autofluorescence under ambient light

**DOI:** 10.1038/s41377-018-0098-6

**Published:** 2018-11-28

**Authors:** Ruikang Zhang, Raja Chouket, Marie-Aude Plamont, Zsolt Kelemen, Agathe Espagne, Alison G. Tebo, Arnaud Gautier, Lionel Gissot, Jean-Denis Faure, Ludovic Jullien, Vincent Croquette, Thomas Le Saux

**Affiliations:** 1 PASTEUR, Département de Chimie, École Normale Supérieure, PSL University, Sorbonne Université, CNRS, 75005 Paris, France; 2 Institut Jean-Pierre Bourgin, INRA, AgroParisTech, CNRS, Saclay Plant Science (SPS), Université Paris-Saclay, Versailles, France; 3 Laboratoire de Physique Statistique, École Normale Supérieure, PSL Research University, Université Paris Diderot Sorbonne Paris-Cité, Sorbonne Université, CNRS, 75005 Paris, France; 4 Institut de biologie de l’École normale supérieure (IBENS), École Normale Supérieure, CNRS, INSERM, PSL Research University, 75005 Paris, France

## Abstract

Macroscale fluorescence imaging is increasingly used to observe biological samples. However, it may suffer from spectral interferences that originate from ambient light or autofluorescence of the sample or its support. In this manuscript, we built a simple and inexpensive fluorescence macroscope, which has been used to evaluate the performance of Speed OPIOM (Out of Phase Imaging after Optical Modulation), which is a reference-free dynamic contrast protocol, to selectively image reversibly photoswitchable fluorophores as labels against detrimental autofluorescence and ambient light. By tuning the intensity and radial frequency of the modulated illumination to the Speed OPIOM resonance and adopting a phase-sensitive detection scheme that ensures noise rejection, we enhanced the sensitivity and the signal-to-noise ratio for fluorescence detection in blot assays by factors of 50 and 10, respectively, over direct fluorescence observation under constant illumination. Then, we overcame the strong autofluorescence of growth media that are currently used in microbiology and realized multiplexed fluorescence observation of colonies of spectrally similar fluorescent bacteria with a unique configuration of excitation and emission wavelengths. Finally, we easily discriminated fluorescent labels from the autofluorescent and reflective background in labeled leaves, even under the interference of incident light at intensities that are comparable to sunlight. The proposed approach is expected to find multiple applications, from biological assays to outdoor observations, in fluorescence macroimaging.

## Bio-imaging: switching signals for clearer fluorescence

Fluorescence imaging at the macroscale will become easier and more sensitive thanks to a new optical procedure, which uses photoswitchable fluorescent tags. Until now, observing signals from the fluorescent labels has been hampered by interference due to external light or the natural fluorescence of other chemicals in a biological sample or in the surrounding media. Thomas Le Saux and colleagues at the École Normale Supérieure, in Paris, with co-workers elsewhere in France, overcame the current limitations using fluorescent labels that can be switched between bright “on” and dark “off” states. Improvements in light exposure and processing methods, combined with this unique characteristic of the label molecules, achieved greatly increased sensitivity in identifying the labels and distinguishing their fluorescence from interfering signals such as autofluorescence or ambient light.

## Introduction

Macroimaging has become an essential tool for studying various types of samples of biological origin (from plate readers, microorganism and cell cultures, tissues, and organs, up to plants and animals). Among the observables, fluorescence has proven particularly attractive due to its sensitivity^1^ and the possibility to fluorescently tag many biomolecules of interest with absolute specificity via genetic fusion^2,3^. Despite these advantages, macroscale fluorescence imaging has several limitations in biological applications. First, many samples exhibit significant background fluorescence, which can originate from the samples themselves (due to autofluorescence or spectrally interfering fluorescent labels), the vessels and substrates, or the imaging media (due to additives such as nutrients and drugs). This can result in a loss of sensitivity, or can impede multiplexed imaging, which is often claimed as an advantage of fluorescence over other observables such as chemiluminescence. In addition, macroscale fluorescence imaging can suffer from ambient light (up to sunlight in the case of outdoor observations); thus, its application is typically restricted to light-tight enclosures or darkrooms. In macroscale fluorescence imaging, the utilization of modulated illumination and image acquisition in homodyne detection^4,5^ and pulsed excitation and time-gated detection^6,7^ has been already proposed for retrieving fluorescence signals against ambient light. In contrast, there remains a demand for reliable strategies for eliminating the detrimental effects of autofluorescence in this imaging modality.

In fluorescence bioimaging, spectral discrimination has been typically favored for generating the contrast of a fluorescent label against an interfering background. Various spectral properties have correspondingly been sought. Hence, labels with (near-infra)red emission have been often favored since the autofluorescence of biological media is generally less pronounced in this wavelength range. However, the corresponding narrow wavelength window intrinsically limits the opportunities for multiplexed observations. Alternatively, spectral discrimination of autofluorescence has been realized with fluorescence unmixing protocols^8,9^; however, this relied on the assumption that a homogeneous spectrum is exhibited over the whole sample. Engaging a label in reactions provides a complementary dimension for overcoming the limitations that are encountered with spectral discrimination. In particular, the dynamic response of a fluorescent label to light modulation has been exploited since kinetics brings as many discriminative dimensions as the number of rate constants involved in the label reactions^1^. This discriminative strategy was explored early in fluorescence spectroscopy, in which the signal originates from the relaxation of an excited label after light absorption by its ground state. Hence, the lifetimes of excited states have been extensively used to discriminate spectrally overlapping labels^10^. However, except in specific favorable cases^11^, the range of lifetimes of the fluorescent labels is narrow (a few nanoseconds) and mostly overlaps the lifetime range of the components that give rise to autofluorescence; thus, deconvolutions^8,12^ or subtractions^13^ must be performed to extract individual contributions, which is detrimental to the signal-to-noise ratio.

**Fig. 1:**
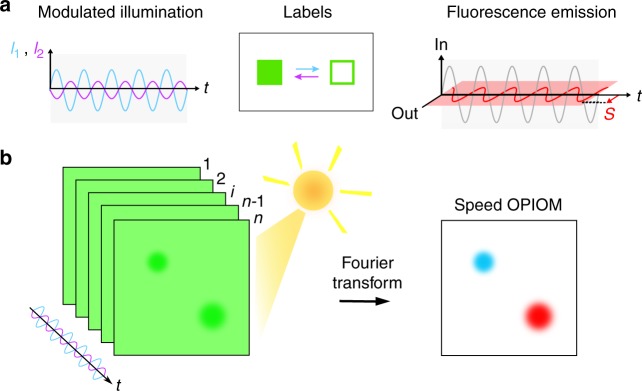
Principle of Speed OPIOM. **a** Sinusoidally modulated light sources that are synchronized in antiphase at two wavelengths generate the quadrature-delayed component *S* (in red) of the fluorescent emission from the reversibly photoswitchable fluorescent labels that are used for selective Speed OPIOM imaging; **b** A dynamic video is recorded upon modulated illumination and the series of images is demodulated to extract *S* from a complex background in the presence of spectrally interfering autofluorescence and ambient light

Reversibly photoswitchable fluorescent labels, which can switch between an ON (bright) state and an OFF (dark) state^14–19^, do not suffer from this drawback since their photoswitching relaxation times cover a much broader range than fluorescence lifetimes. For instance, we recently showed that the photoswitching relaxation time of genetically encoded green-emissive reversibly photoswitchable fluorescent proteins (RSFPs) spans over three orders of magnitude^20^. Hence, several microscopy protocols (e.g., optical lock-in detection (OLID)^21^, synchronously amplified fluorescence image recovery(SAFIRe)^22^, and out-of-phase imaging after optical modulation, which is denoted as OPIOM^20,23,24^) have been recently introduced for kinetically discriminating reversibly photoswitchable fluorescent labels against several types of spectrally interfering backgrounds without a deconvolution or subtraction scheme. In OPIOM^20,23,24^, kinetic information of a targeted fluorescent label is uncovered by driving its reversible photoswitching between states of contrasted brightness. Subsequent filtering enables one to selectively extract the contribution of this label from the overall signal, even in the presence of interfering species. More specifically, Speed OPIOM drives photoswitching with two modulated light sources that are synchronized in antiphase at two wavelengths (Fig. 1a). After the acquisition of tens to hundreds of images for at least two periods of light modulation, Speed OPIOM signal *S*—the out-of-phase component of the modulated fluorescence signal—is directly retrieved via Fourier transform of the acquired images (Fig. 1b); no further processing is needed and lock-in detection ensures efficient noise rejection. *S* exhibits a narrow resonance when the light intensities are tuned to balance the average concentrations of the ON and OFF states (to maximize the photoswitching amplitude at the changes of light intensities) and the angular frequency is matched with the inverse of the photoswitching relaxation time^20^ (to generate the phase lag that optimizes the out-of-phase amplitude of the fluorescence response; see ref. ^20^ for a complete description). Since a reversible photoswitchable fluorescent label has unique photochemical and kinetic properties (and, correspondingly, its own resonance conditions), simply tuning the illumination control parameters to the resonant values enables one to selectively image a targeted label, filtering out the contributions of nonresonant fluorophores and ambient light (Fig. 1b).

In the present manuscript, we implement Speed OPIOM in macroscale fluorescence imaging. We build a simple and cheap macroscope and thoroughly evaluate its efficiency in eliminating the interference of autofluorescence and ambient light when observing targeted RSFPs in three types of biologically relevant samples. First, we examine its application to improve the sensitivity of blot assays. Most commercially available porous membranes that are used for protein transfer (nitrocellulose, polyvinylidene difluoride) emit a significant fluorescence in the blue–yellow wavelength range of the visible spectrum, which worsens the analytical performance after revelation with fluorescently labeled antibodies^25–28^. We demonstrate that our macroscope enhances the sensitivity and signal-to-noise ratio for fluorescence detection on nitrocellulose membranes by factors of 50 and 10, respectively, over direct fluorescence observation under constant illumination. Then, we apply our setup to the imaging of colonies and lawns of fluorescent bacteria that are growing on agar plates. Fluorescence plates are widely used to analyze the level of protein production^29^ and the organization of microbial biofilm communities^30^ and to screen for compounds that select against antibiotic resistance^31^; however, they often suffer from a significant autofluorescence that originates from the plate material or the growth media^32^. We demonstrate that our macroscope overcomes the strong autofluorescence of the growth media that are used in microbiology and enables multiplexed fluorescence observation with the same configuration of excitation and emission wavelengths. Finally, we address macroscale fluorescence imaging of fluorescently labeled leaves under strong external illumination. Leaves are strongly reflective and autofluorescent heterogeneous media in which macroimaging of fluorescent proteins has been hampered by endogenous fluorophores^33,34^ (e.g., the photosynthetic apparatus) and has necessitated sophisticated time-gated detection schemes^35^. We demonstrate that our setup for macroscale imaging easily discriminates fluorescent labels from the autofluorescent background, even at light intensities that are comparable to sunlight.

## Results

### Speed OPIOM macroscope

**Fig. 2:**
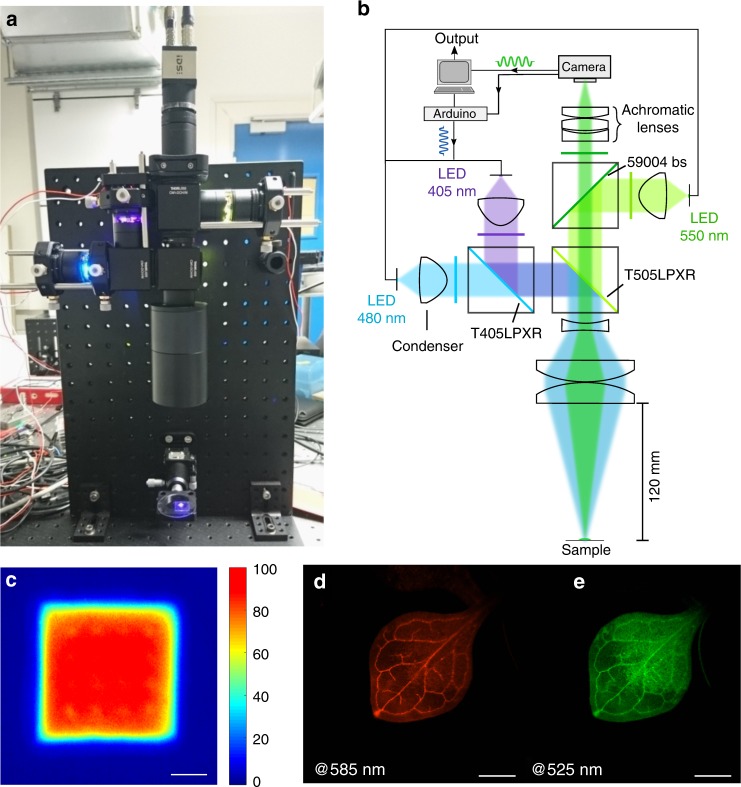
Presentation of the Speed OPIOM macroscope. **a** The optical setup for Speed OPIOM macroscale fluorescence imaging. **b** A schematic diagram of the setup, which shows the optical layout and the interface to a PC-based synchronized modulation and image acquisition system. **c** The normalized light illumination is homogeneous at the sample. **d**, **e** Chromatic correction yields sharp images of leaves of Dronpa-2 and DsRed-double-labeled *Arabidopsis thaliana* in both the red (585 nm) and green channels (525 nm). Scale bars: 1 mm

Speed OPIOM imaging requires well-controlled illumination homogeneity to enable selective imaging of a targeted reversibly photoswitchable fluorophore over the whole image. More precisely, the average intensities of the light sources, which are denoted as $I_{1}^{0}$ and $I_{2}^{0}$ and modulated at angular frequency *ω* must be kept constant over the whole imaged field since the Speed OPIOM resonance conditions fix both $I_{1}^{0}/I_{2}^{0}$ and $I_{1}^{0}/{\omega}$^20^. Additionally, one should adopt powerful light sources to record the images at the highest possible acquisition frequency. We have chosen light-emitting diodes (LEDs) as the light sources for our macroscope since they are compact, inexpensive, robust, easy to modulate, and flexible for optical design. Hence, our macroscope integrates three 500 mW colored LEDs with center wavelengths of 405, 480, and 550 nm (Fig. 2a, b). The two LEDs that emit at 405 and 480 nm are directly involved in the implementation of Speed OPIOM with RSFPs (vide infra); the LED that emits at 550 nm has been added to excite red fluorescent proteins in view of agronomic applications to be published elsewhere. After collimation by high-NA condensers and bandpass filtering, the excitation lights are combined by three beamsplitters and refocused by a beam expanding system (which is composed of three optical elements; see Fig. 2b, the electronic supplementary material and Fig. S1) onto the sample, which is located 120 mm away, to generate uniform illumination within a square-shaped area of 4 × 4 mm^2^ with high irradiance for the three excitation wavelengths. The maximum irradiance is estimated to exceed 1000 mW/cm^2^ at each wavelength and the distribution of the light intensity is essentially uniform with a deviation of less than 25% throughout the square (Fig. 2c), which is fully compatible with the Speed OPIOM requirements.

The fluorescence emission is collected by the same optical system and filtered by bandpass filters that are centered at 525 and 585 nm for green and red fluorescent labels, respectively. The image is formed by an achromatic lens system (which is composed of three spherical singlet lenses with optimized air spaces and an effective focal length of *f* = 30 mm; see Fig. 2b, the electronic supplementary material and Fig. S2) onto the camera, which enables us to obtain sharp images for both the green and red channels from 500 to 650 nm (Fig. 2d, e, Figs. S3, S4 and S5). The modulation reference signal and the acquisition of the camera are synchronized by an Arduino-compatible card (Teensy 3.5) and the series of acquired images is subsequently processed by a computer. After preliminary correction of any photobleaching that occurred during the illumination sequence, the time domain Fourier transform is applied to the series of captured images to yield the average and out-of-phase images, which are denoted as Pre-OPIOM and OPIOM, respectively. The Pre-OPIOM image compares well with the fluorescence image, which would be classically recorded under constant illumination.

### Enhancing the contrast and sensitivity in blot analysis

Blots are among the most important analytical tools in cell biology, immunogenetics and other biological fields. Fluorescence has attracted increasing attention for the quantitative measurement of protein amounts since it provides opportunities for multiplexed observations. However, its sensitivity has been limited by the autofluorescence of the transferring membranes^26^: Upon excitation with UV or blue light, these membranes emit significant fluorescence from the blue to the yellow regions of the visible spectrum, which overlaps the emission spectra of the most commonly used fluorescent probes, thereby limiting the reliable detection of targeted proteins to no less than 0.5 ng^36^.

**Fig. 3:**
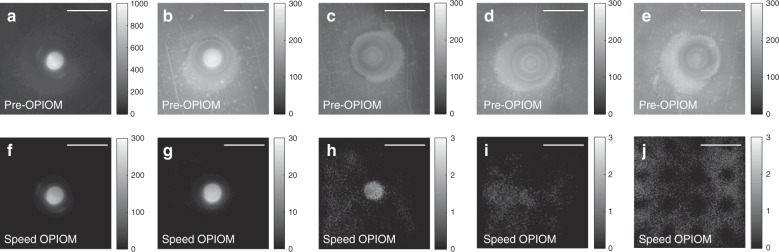
Speed OPIOM to overcome the limitations of autofluorescence in blot analysis. Pre-OPIOM (**a**−**e**) and OPIOM (**f**−**j**) images of the nitrocellulose membrane on which various amounts of Dronpa-2 (in pg) have been percolated within the deposited 400 µm-diameter blot: **a**, **f** 250; **b**, **g** 25; **c**, **h** 2.5; **d**, **i** 0.5; and **e**, **j** 0 (control). The images were captured at resonance for Dronpa-2. The image acquisition conditions are specified in Table S1. Scale bars: 1 mm

To evaluate the ability of our imaging setup to overcome the interference of the membrane autofluorescence and to improve the contrast and the sensitivity of fluorescent blotting, we adopted the 28 kDa Dronpa-2^37,38^ RSFP as the targeted reversibly photoswitchable fluorescent label and a commercially available and widely used nitrocellulose membrane as a representative transferring substrate, on which various Dronpa-2 amounts have been blotted. Figure 3 displays the Pre-OPIOM and OPIOM images of the membrane that were recorded in a dark room. In the Pre-OPIOM images (Fig. 3a−e), the 250 pg blot can be clearly detected and the 25 pg blot is perceptible but has significantly lower contrast; however, the blot cannot be observed below 5 pg against the overwhelming membrane autofluorescence. In contrast, the blot is reliably observed in OPIOM images down to an amount of 2.5 pg with excellent contrast and image resolution due to total elimination of the autofluorescence (Fig. 3f−j).

**Fig. 4:**
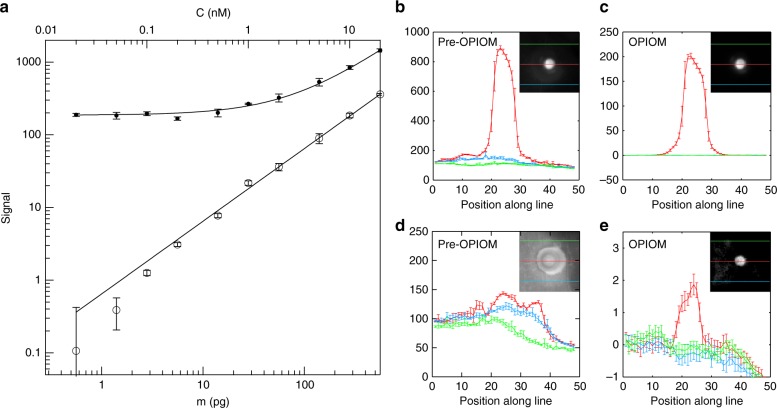
Speed OPIOM enhancement of contrast and sensitivity in blot analysis. Dependence of the mean signal (**a**) and of the signal profiles (**b–e**) on the amount of Dronpa-2 in the Pre-OPIOM and OPIOM images. **a** Pre-OPIOM (disk) and OPIOM (circle); the error bar indicates the standard deviation over three independent measurements. **b**, **c** 250 pg, **d**, **e** 2.5 pg. In (**b–e**), the analyses of both Pre-OPIOM and OPIOM have been performed along three lines: one crosses the middle of the blot (displayed in red), whereas the two others (shown in green and blue) are located outside of it (see also Fig. S6)

We further analyzed the collected Pre-OPIOM and OPIOM images to quantitatively estimate the contrast and sensitivity improvements that were realized by applying the Speed OPIOM protocol in blot analyses. We computed the values of the mean signals and the standard deviations in each 5 × 5 pixel^2^ area and subsequently computed the signal-to-noise ratios. The analyses have been performed along three lines: one crosses the middle of the blot and the two others are located on the homogeneous membrane area away from the first line as controls (see Fig. S6). Figure 4a displays the dependence on the Dronpa-2 amount of the average intensity at the blotted spot in both the Pre-OPIOM and OPIOM images. In Pre-OPIOM, the dependence is linear down to 25 pg. Below this amount, the membrane autofluorescence dominates the signal, thereby impeding the reliable quantification of the Dronpa-2 content. In contrast, OPIOM restores the linearity between the signal and the amount of Dronpa-2 over the entire 500–2.5 pg range. This linearity is still observed down to 0.5 pg, but only for the average signal over three measurements.

The signal-to-noise ratios of the Pre-OPIOM and OPIOM images of the 250 pg blot are respectively equal to 30 and 800 based on an estimate of the background contribution over a homogeneous area on the membrane (Fig. 4b, c). The large improvement of the signal-to-noise ratio that is realized by the OPIOM protocol is the result of filtering out a large amount of the shot noise via lock-in demodulation and fluorescent background annihilation providing zero average contribution of the membrane to the OPIOM signal. The Pre-OPIOM signal-to-noise ratio could not be reliably evaluated at 2.5 pg since the observed signal of the blotted Dronpa-2 was lower than the standard deviation over the autofluorescent membrane (Fig. 4d; see also the control in Fig. S6, where only the buffer has been deposited on the membrane). In contrast, the 2.5 pg deposit of Dronpa-2 was clearly observable in the OPIOM image with a signal-to-noise ratio of 6 (Fig. 4e).

According to these observations, our macroscope can selectively extract the signal of interest and enhance the sensitivity of fluorescent blotting by 1–2 orders of magnitude and the signal-to-noise ratio by at least a factor of 10 over the entire relevant concentration range (see Fig. S6).

### Multiplexed macroscale fluorescence imaging

**Fig. 5:**
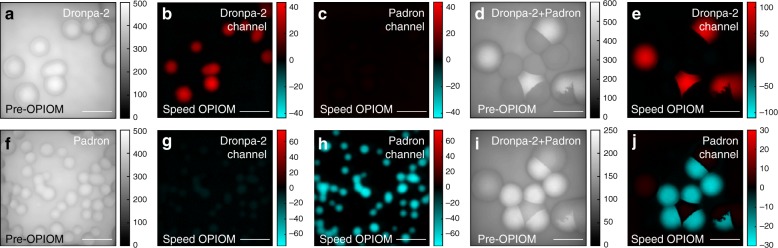
Speed OPIOM for multiplexed macroscale fluorescence imaging. Pre-OPIOM (**a**, **d**, **f**, **i**) and Speed OPIOM (**b**, **c**, **e**, **g**, **h**, **j**) images of *E. coli* bacteria that are growing on agar plates and expressing Dronpa-2 (**a–c**), Padron (**f–h**) or a coculture of *E. coli* bacteria that is expressing Dronpa-2 or Padron (**d**, **e**, **i**, **j**). The images were recorded at resonances of Dronpa-2 (**a**, **b**, **d–g**) and Padron (**c**, **h**, **i**, **j**). The image acquisition conditions are specified in Table S1. Scale bars: 1 mm

Beyond eliminating autofluorescence, Speed OPIOM is compatible with the discrimination of spectrally similar reversibly photoswitchable fluorescent labels, which exhibit distinct photoswitching dynamics^20^. To illustrate this attractive feature for multiplexed macroscale fluorescence observations, we imaged colonies of *E. coli* bacteria that each expressed one of two green fluorescent RSFPs, namely, Dronpa-2^37,38^ and Padron^39^, and were growing on a strongly autofluorescent growth medium (Fig. 5a−j). Again, we could efficiently eliminate the strong autofluorescent background that arises from the solid growth medium. Moreover, since the RSFPs exhibit Speed OPIOM resonances for different values of the illumination parameters, we could selectively retrieve their individual fluorescence contributions. At resonance for Dronpa-2, Fig. 5b, e shows that the Dronpa-2-labeled bacteria exhibit a strong OPIOM signal, whereas the signal from the Padron-labeled bacteria is turned off. In contrast, at resonance for Padron, Fig. 5h, j shows the Padron-labeled bacteria without any interference from the Dronpa-2-labeled bacteria. Interestingly, discrimination is additionally facilitated by the opposite signs of the algebraic Speed OPIOM observables for Dronpa-2 and Padron, which are governed by the opposite signs of their photochromisms.

### Fluorescence imaging under sunlight conditions

Fluorescence is used to image not only cells but also increasingly whole living organisms such as plants^35^ and animals^40^. For this type of observations, ambient light is always interfering and degrades the contrast and the sensitivity of the acquired images. In particular, although fluorescence imaging techniques against adverse lighting conditions such as room light or shaded sunlight have been proposed^6^, fluorescent detection under strong direct sunlight in outdoor conditions has not yet been reported.

**Fig. 6:**
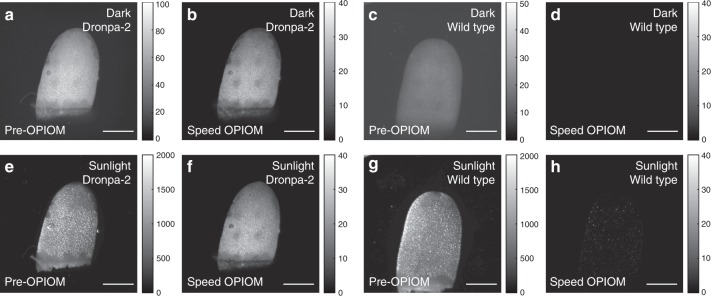
Speed OPIOM for fluorescence imaging under sunlight conditions. Pre-OPIOM (**a**, **c**, **e**, **g**) and Speed OPIOM (**b**, **d**, **f**, **h**) images of Dronpa-2-expressing (**a**, **b**, **e**, **f**) and wild-type (**c**, **d**, **g**, **h**) *Camelina Sativa* in the darkroom (**a–d**) and under artificial sunlight (**e–h**) conditions. The image acquisition conditions are specified in Table S1. Scale bars: 1 mm

Unlike cells or animal tissues, which are transparent or semitransparent, plant leaves reflect approximately 5−20% of the sunlight in the visible wavelength range^41^, which makes them especially difficult to image with fluorescence macroscopy; in particular, 15–20% of the incident light is reflected at approximately 525 nm, thereby making it more difficult to shade the reflected sunlight, even with a narrow bandpass filter that is designed for green fluorescence. To evaluate the performance of our setup for accurate fluorescent imaging of plants under direct sunlight, we imaged Dronpa-2-expressing *Camelina sativa* leaves using wild-type plants as controls. To make the experiment even more quantitative, we used an LED to simulate the interference of the sunlight in the wavelength range of the emitted RSFP fluorescence that was collected by our macroscope. Figure 6 displays images of leaves that were recorded in the dark and under artificial sunlight conditions that match the optical power that is sent to the sample under real sunlight. In the dark (Fig. 6a−d), both the wild-type and Dronpa-2-expressing leaves are clearly observed in the Pre-OPIOM images, which demonstrates the significant autofluorescence emission of the sample; in contrast, Speed OPIOM provides a nonvanishing signal only for the leaf of the Dronpa-2-expressing plant. When the leaves are exposed to artificial sunlight (Fig. 6e−h), the Pre-OPIOM signals of both the wild-type and Dronpa-2-expressing leaves are approximately 20 times stronger than in the dark, where autofluorescence is ubiquitously detected (Fig. 6a, c): sunlight reflection on the leaf epiderm dominates any fluorescence signal. In contrast, the OPIOM images (Fig. 6f, h) retain the Dronpa-2 signal of the Dronpa-2-expressing leaf that is measured in the darkroom. Hence, our macroscope provides accurate information on the fluorescence signal of the Dronpa-2 label without any interference from ambient light, even at high intensity.

## Discussion

The preceding series of experiments has demonstrated the performance of the Speed OPIOM protocol, which was implemented in our simple optical setup, in overcoming the limitations of spectrally interfering (auto)fluorescence and ambient light in fluorescence macroscale imaging. In the following paragraphs, the scope and limitations with respect to the fluorescent labels to be used and the maximal achievable contrast against spectral interferences are discussed.

In the present account, we relied on RSFPs for the experimental validations. Numerous categories of fluorophores exhibit photochemical behaviors that can be exploited by our setup to broaden the application scope of the OPIOM protocols. In particular, numerous molecular backbones exhibit reversible photoswitching when they are exposed to light^16,19,42^. When the backward isomerization that follows forward photoswitching is thermally driven, one can use the original OPIOM protocol, which exploits the resonant modulation of monochromatic light, to eliminate spectral interferences^23^. Alternatively, when the forward and backward photoswitching processes are photochemically governed at two distinct wavelengths, as in many reversibly photoswitchable labels (e.g., RSFPs^17,18^, azobenzenes^43–45^, cyanines^14,46,47^, diarylethenes^15,48^, and spirobenzopyrans^49^), one should preferentially rely on the Speed OPIOM protocol that is used in this study since it gives rise to an amplitude that is twice that of the collected signal and, accordingly, a higher signal-to-noise ratio. Our approach can also be applied to nonphotoswitching fluorophores if their photophysical scheme can be dynamically reduced to a two-state model on a sufficiently long time scale. We have already shown that OPIOM can be adapted to three-state models that involve a reaction in the excited state (e.g., intersystem crossing)^23^.

OPIOM application requires the tuning of the control parameters of illumination (the angular frequency of light modulation and the average photon flux per unit of surface at the sample) to match the resonance conditions of the targeted fluorophore. In OPIOM, the resonant angular frequency only depends on the rate constant for thermal relaxation after photoswitching^23^.  In contrast, it can be tuned with more flexibility in the two-color Speed OPIOM approach: one can increase it by increasing the mean intensities of both light sources if their ratio remains constant. The nature of the light sources to be used depends on the time scale at which the scheme for reversible fluorophore photoactivation can be reliably reduced to a two-state model and on the sample distance: both a shorter time scale and a larger distance necessitate higher light powers. Since the relevant reversibly photoswitchable fluorescent labels^14–19^ exhibit photoswitching relaxation times that are in the µs–s range, easily modulatable LEDs could be often used as excitation light sources to meet the resonance conditions in fluorescence macroscopy at 0.1–1 m imaging distance; modulatable lasers should be used at longer imaging distances. In principle, it is advantageous to adopt the highest possible value of the angular frequency of light modulation (and, correspondingly, light sources with the highest possible intensity) to shorten the measurement duration (which must be at least two periods of light modulation) and avoid noise (which is typically more abundant at low frequencies). Since the delivered irradiance is higher than 1000 mW/cm^2^ at both the 405 and 480 nm wavelengths, which substantially exceeds the maximum solar radiation (≈10 mW/cm^2^ for 40 nm bandwidth at both excitation wavelengths), our optical design can drive and image the photoswitching of Dronpa-2 at a Speed OPIOM resonance of up to 3 Hz with full amplitude modulation. The photoswitching features of Dronpa-2 enable its Speed OPIOM imaging up to a modulation frequency of 50 Hz^20^ by focusing light that is emitted from the LEDs over a smaller area or using more powerful light sources such as lasers.

In terms of the elimination of autofluorescence and ambient light, our setup suffers from two intrinsic limitations. Our first limitation arises from the limited phase retrieval precision when extracting the out-of-phase component of the fluorescence modulation. Typically, we rely on preliminary calibrations to retrieve the phase information by analyzing the response of the attenuated light source or of a fast-responding fluorophore (e.g., fluorescein). Alternatively, one could tune the phase lag for signal detection to eliminate the contribution of autofluorescence or ambient light under the experimental conditions. Any error in phase retrieval will introduce a contribution from the in-phase component of the fluorescence modulation, which contains the interfering contributions of autofluorescence and ambient light. With a present precision, which is in the 3×10^−3^ rad range, we could typically enhance the contrast of the targeted fluorophore against the spectrally interfering signal by a factor 10^2^−10^3^. The second limitation originates from the enhanced and intrinsic noise of the detector. Indeed, any strong signal from the spectrally interfering species will generate a photon noise that interferes with the extraction of the OPIOM signal. For instance, the minimal OPIOM signal that is detectable against ambient light scales as the square root of the photon flux of the ambient light^20^, which typically enables one to detect the OPIOM signal, even in the presence of a 10^3^-time-stronger nonmodulated photon source.

Finally, we underline the relatively low cost (less than 10k €; see the electronic supporting material) of our prototype, which consists only of standard optical equipment and a camera for industrial vision. This cost could be optimized by using the reflecting properties of the bandpass filters and a special arrangement of LED chips would enable the combination of the lights from the colored LEDs with a single dichroic mirror for short-range imaging (0.1−1 m). For Speed OPIOM imaging at longer distances, modulatable lasers (approximately 6k € per color) should be used. Eventually, due to rapidly improving performance (in terms of lower dark and read noises, higher quantum yield, and higher saturation capacities), the next generation of the complementary metal oxide semi-conductor (CMOS) sensors that are integrated in such cameras (including the backside illumination configuration) should enable even better performance to be realized in terms of the signal-to-noise ratio and the dynamic range.

In conclusion, this study has demonstrated the ability of a simple and cheap fluorescence macroscope to overcome the detrimental limitations of autofluorescence and strong ambient light for macroimaging. Beyond this achievement, this setup, combined with a powerful reference-free dynamic contrast protocol, substantially enhances the sensitivity and the signal-to-noise ratio for fluorescence detection. Compatible with a large collection of fluorophores, it opens up additional opportunities for multiplexed fluorescence observations by overcoming the limitations that are encountered with spectral discrimination. Thus, this macroscope is expected to find multiple applications in diverse areas such as quantitative bioanalysis and diagnostics (multiplexed western blots, enzyme linked immunosorbent assays (ELISAs), and cytometry), bioassays, microorganism selection, plant research, and environmental monitoring.

## Materials and methods

### Setup for macrofluorescent imaging

#### Excitation system

Our setup uses three high-power color LED chips (LXZ1-PB01, LHUV-0400, and LXZ1-PX01; Lumileds, NL) as excitation lights for the green (such as the RSFPs that are used in this work) and red (such as DsRed, which is subsequently used in an application development) fluorescent emitters. The sources are collimated by high-NA condensers (ACL25416U-A, *f* = 16 mm, Thorlabs, NJ, USA) and filtered by bandpass filters (ET470/40×, ET402/15×, ET550/15×; Chroma, VT, USA) to avoid spectral overlaps. The three quasi-parallel beams are combined by three dichroic mirrors (T425LPXR, T505LPXR, and 59004bs; Chroma, VT, USA). An optimized beam expander system that integrates one divergent lens (ACN254-040-A, *f* = −40 mm, Thorlabs, NJ, USA) and two convergent lenses (AC508-100-A, *f* = 100 mm, Thorlabs, NJ, USA) is used to clearly refocus the lights at a distance of 120 mm onto the sample (see the electronic supplementary material).

#### Imaging system

To obtain the fluorescence image of the illuminated area, the fluorescent light that is emitted from the sample is collected by the expander system and imaged to infinity. A bandpass filter (ET525/36m or ET585/20m, Chroma, VT, USA) is used for green emission or red emission. Then, light is focused onto the camera (iDS 3060cp), which captures an image of the fluorescent sample. As the detectable sample is the same size as the illumination area (4 × 4 mm^2^), an extra lens system is designed for the macroimaging of small size objects, which requires high-resolution and well-corrected chromatic aberration from red to green, which is a spectral range that cannot be corrected by a regular lens or even an achromatic lens. The elaborate achromatic system (see Fig. 2), which has an effective focal length of *f* = 30 mm, is composed of three spherical singlet lenses (LC2679-A, LB1757-A, and LA1422-A, Thorlabs, NJ, USA) and the air spaces are optimized (see the electronic supplementary material).

#### Video acquisition

In the imaging experiments, we record films for *m* periods of light modulation (*m* is an integer). The acquisition frequency of the camera is set such that 2*N* (*N* is an integer) frames are obtained per period of modulation. Thus, the acquisition frequency is *f*_s_ = 2*Nf*_m_, where *f*_m_ is the modulation frequency of the excitation light. The fluorescence emission that is acquired at pixel (*x*,*y*) of the *k*th frame is equal to

1 $${{I_{\rm{F}}\left( {x,y,k} \right)} = T_{\rm S}\left\{\Im _{\rm{F}}^{0}\left( {x,y} \right) + \mathop {\sum }\limits_{n = 1}^{N} \left\{ \Im _{\rm{F}}^{n,{\rm{sin}}}\left( {x,y} \right)\sin \left[ {\frac{{\pi nf_{\rm s}}}{N}\left( {\frac{k}{{f_{\rm s}}}} \right) + \phi _{{\rm{acq}}}} \right] \right. \right.} \\ {\hskip 10pt \left. \left. + \, \Im _{\rm{F}}^{n,{\rm{cos}}}\left( {x,y} \right)\cos \left[ {\frac{{\pi nf_{\rm s}}}{N}\left( {\frac{k}{{f_{\rm s}}}} \right) + \phi _{{\rm{acq}}}}\right]\right\}\right\}}$$

where $T_{\mathrm S} = \frac{1}{{f_{\mathrm s}}}$ refers to the exposure time of a single frame; and $\Im _{\mathrm{F}}^{n,{\mathrm{cos}}}\left( {x,y} \right)$ are the sinus and cosinus components, respectively, of the fluorescence signal at harmonic *n* around the average value $\Im _{\mathrm F}^0$; and *ϕ*_acq_ is a time lag, which may originate from the difference in starting times between the light modulation and the acquisition of the camera.

Preprocessing has been performed over the video to compensate for possible photobleaching of the fluorophores. By assuming that the photobleaching exhibits a linear decay, the compensation factor is calculated from the average of two successive periods:

2 $$K\left( {x,y} \right) = \frac{{I_{\mathrm{F}}(x,y,k)_{k = 2N}^{4N - 1} - I_{\mathrm{F}}(x,y,k)_{k = 0}^{2N - 1}}}{{2N}}$$

Then, all the frames over the two periods are corrected via Eq. (3):

3 $$I_{\mathrm{F}}^{{\mathrm{corr}}}(x,y,k) = I_{\mathrm{F}}\left( {x,y,k} \right) - K\left( {x,y} \right) \times k$$

All the frames over the whole video have been corrected for photobleaching by applying the same algorithm for all successive pairs of periods.

*ϕ*_acq_ can be easily calibrated by using the fluorescence emission from instantaneously responding fluorophores (such as EGFP or Fluorescein). The Pre-OPIOM image is calculated by averaging the frames over the whole film and normalizing to unit time to yield $\Im _{\mathrm{F}}^0\left( {x,y} \right)$:

4 $$\Im _{\mathrm{F}}^0\left( {x,y} \right) = \frac{{f_{\mathrm s}}}{{2mN}}\mathop {\sum }\limits_{k = 0}^{2mN - 1} I_{\mathrm{F}}^{{\mathrm{corr}}}(x,y,k)$$

Demodulation is performed by multiplying the *k*th frame, which is denoted as $I_{\mathrm{F}}^{{\mathrm{corr}}}(x,y,k)$, with $\cos \left[ {\frac{{\pi nf_{\mathrm s}}}{N}\left( {\frac{k}{{f_{\mathrm s}}}} \right) + \phi _{{\mathrm{acq}}}} \right]$and averaging over the whole film to obtain the first-order cosinus component, namely, the Speed OPIOM image:

5 $$\Im _{\rm{F}}^{1,{\rm{cos}}}(x,y) \\ 	 \hskip -5pt = \frac{{f_{\rm s}}}{{2mN}}\mathop {\sum }\limits_{k = 0}^{2mN - 1} \left\{ {I_{\rm{F}}^{{\rm{corr}}}(x,y,k) \times \cos \left[ {\frac{{\pi nf_{\rm s}}}{N}\left( {\frac{k}{{f_{\rm s}}}} \right) + \phi _{{\rm{acq}}}} \right]} \right\}$$

#### Calibration of the light intensity

Speed OPIOM implementation requires the intensities of the two excitation lights at the sample to be determined (typically at 480 and 405 nm) to satisfy the resonant illumination conditions for the desired RSFPs. Instead of using a powermeter for our intensity measurements, we preferred to directly exploit the dynamical photochemical properties of the RSFPs. The main objective of the calibration experiments is to measure the relaxation time that is associated with the conversion between the ON and OFF RSFPs states by applying light jumps on an RSFP-containing sample. We typically used a thin layer of RSFP solution that was spread on the coverslip as the calibrating sample. In the first step, the sample was subjected to a light jump at 480 nm with constant light intensity $I_1^0$, in which the current of the LED source was set to approximately 500 mA to yield the maximal power. Upon illumination, the RSFP switched from its thermodynamically stable state (denoted as **1**) to its photoisomerized state (denoted as **2**) and the fluorescence image was recorded as a function of time. Then, the fluorescent signal (which was obtained by averaging over the image) was plotted as a function of time (Figs. S7a, S8a) and fitted with Eq. (6):

6 $$I_{\mathrm{F}}\left( t \right) = I_{\mathrm{F}}\left( {0,{{\lambda }}_1} \right) + {{{A}}}_{{{\lambda }}_1}\left[ {1 - {\mathrm{exp}}\left( { - \frac{{{t}}}{{\tau _{{{\lambda }}_1}}}} \right)} \right]$$

where ${{{A}}}_{{{\lambda }}_1}$ is a pre-exponential term that accounts for the molecular brightnesses of the ON and OFF states and their relative proportions (see ref. ^20^), $\tau _{\lambda _1}$ was extracted from the fit and $I_1^0$ was subsequently obtained via Eq. (7):

7 $$\frac{1}{{\tau _{\lambda _1}}} = k_{21}^{\mathrm{\Delta }} + \left( {\sigma _{12,\lambda _1} + \sigma _{21,{\mathrm{\lambda }}_1}} \right)I_1^0$$

where $\sigma _{12,\lambda _1}$ and $\sigma _{21,\lambda _1}$ are the molecular action cross-sections for photoisomerization from **1** to **2** and **2** to **1**, respectively (see ref. ^20^). In the second step, while $I_1^0$ was maintained at its original value, the sample was subjected to a light jump at 405 nm at constant light intensity $I_2^0$. A reverse switch took place and the temporal evolution of the fluorescence emission was again recorded (Figs. S7b, S8b) and fitted with Eq. (9):

8 $$I_{\mathrm{F}}\left( t \right) = I_{\mathrm{F}}\left( {0,{{\lambda }}_1,{{\lambda }}_2} \right) + {{{A}}}_{{{\lambda}}_1{{\lambda}}_2}\left[ {1 - {\mathrm{exp}}\left( { - \frac{{{t}}}{{{{\tau }}_{{{\lambda}}_1{{\lambda}}_2}}}} \right)} \right]$$

where ${{{A}}}_{{{\lambda}}_1{{\lambda}}_2}$ is a pre-exponential term that accounts for the molecular brightnesses of the ON and OFF states and their relative proportions (see ref. ^20^), ${{\tau}}_{{{\lambda}}_1{{\lambda}}_2}$ was extracted from the fit and $I_2^0$ was subsequently obtained via Eq. (9):

9 $$\frac{1}{{\tau _{{{\lambda }}_1{{\lambda }}_2}}} = k_{21}^{\mathrm{\Delta }} + \left( {\sigma _{12,{{\lambda }}_1} + \sigma _{21,{{\lambda }}_1}} \right)I_1^0 + \left( {\sigma _{12,{{\lambda }}_2} + \sigma _{21,{{\lambda }}_2}} \right)I_2^0$$

where $\sigma _{12,{{\lambda }}_1} + \sigma _{21,{{\lambda }}_1}$and $\sigma _{12,{{\lambda }}_2} + \sigma _{21,{{\lambda }}_2}$ are the molecular action cross-sections for photoisomerization at wavelengths ${{\lambda }}_1$ and ${{\lambda }}_2$, respectively, from **1** to **2** and **2** to **1**. After repeating this experiment for several values of $I_1^0$ and $I_2^0$, we used the reported values of $\sigma _{12,{{\lambda }}_1} + \sigma _{21,{{\lambda }}_1}$=196 m^2^/mol and $\sigma _{12,{{\lambda }}_2} + \sigma _{21,{{\lambda }}_2}$ = 413 m^2^/mol for Dronpa-2 ^20^ to finalize the calibration that links the current intensities that are applied to the LEDs with $I_1^0$ and $I_2^0$.

### Speed OPIOM macroimaging

#### Blot samples

##### Image recording

The Dronpa-2-blotted membrane was placed on a cover slip and put on the focal plane under the macroimaging device. The measurement was performed at 24 °C while the blotted area was still wet. LED lights at 480 nm ($I_1^0$ *=* 4×10^−2^ ein/m^2^/s) and 405 nm ($I_2^0$ = 1.9×10^−2^ ein/m^2^/s), that were synchronized in antiphase and sinusoidally modulated at 2.5 Hz were applied to irradiate the membrane and a series of fluorescence images of the membrane was simultaneously captured by the CCD camera with a sampling rate of $f_{\mathrm s}$ = 50 Hz. For each measurement in this series of experiments, the excitation lights were modulated for eight cycles, thereby leading to the capture of 160 images in less than 4 s. For each Dronpa-2 concentration, three samples were independently measured to evaluate the reliability of the results.

##### Estimation of the signal-to-noise ratios

The Pre-OPIOM and OPIOM images were averaged over 5 × 5 pixel^2^. Then, we computed the mean and standard deviation values for each pixel. We subsequently chose three lines in the pixelized Pre-OPIOM and OPIOM images (indicated by the colored lines in Fig. 4 and Fig. S6): a first line that crosses the area where Dronpa-2 was deposited and two additional lines above and below the deposition area, where the fluorescence background was at its most homogeneous. The maximum value, which is denoted as $S_{{\mathrm D}2}$, in the blot area of Dronpa-2 was obtained from the first profile line. The mean value ${\mathrm {bg}}$ and standard deviation $\sigma _{{\mathrm {bg}}}$ along the two other profile lines were calculated to estimate the background contributions. The signal-to-noise ratios were estimated as SNR=$\left( {S_{{\mathrm D}2} - {\mathrm {bg}}} \right){\mathrm{/}}\sigma _{{\mathrm {bg}}}$ using data from both profile lines that were obtained in the membrane zone. Eventually, we retained the lowest calculated value as the SNR (see Fig. S6).

##### *E. coli* bacteria on a solid growth medium

The agar plates on which the bacteria grew were placed on the focal plane under the macroimaging setup. The bacteria samples (colonies of bacteria that were labeled with either Dronpa-2 or Padron, or coexisting colonies from both strains) were successively imaged under two illumination conditions that were associated with the resonances of Dronpa-2 and Padron. For Dronpa-2 imaging, we adopted a sampling rate of $f_{\mathrm s}$ = 50 Hz with a light modulation at 5 Hz for eight periods. For Padron imaging, we used a light modulation at 0.02 Hz for two periods at a sampling rate of $f_{\mathrm s}$ = 4 Hz. Imaging was performed in a dark room at 24 °C.

##### Plant leaves

The leaves of *Camelina sativa* were placed on a cover slip that was immersed with water. Light from an LED with a green emission that was centered at 530 nm (LXZ1-PM01) was collimated and delivered to the sample to simulate the interference of sunlight in the wavelength range that is analyzed with our macroscope. To match the optical power that is sent to the sample with the real sunlight power, a photodiode power meter (PM100A, Thorlabs, DE) was used to measure the sunlight and the intensity of the LED on the sample. Since the recorded green fluorescent emission is detected after bandpass filtering (ET525/36m, Chroma, USA) in our setup, we applied the same filter to record the irradiance of the direct sunlight in the same green waveband (525 ± 18 nm). We measured 10 mW/cm^2^ (*λ* *=* 525 nm, Δ*λ* = 36 nm). We correspondingly adjusted the power of the green LED to deliver the same power in our experiments. The images of the leaves have been recorded under both darkroom and artificial sunlight conditions at a sampling rate of $f_{\mathrm s}$ = 200 Hz by modulating the excitation lights at 2.5 Hz for eight periods.

## Electronic supplementary material

Supplementary Material
