# Supplementary material for: Macroscale fluorescence imaging against autofluorescence under ambient light
Source: Light Sci Appl. 2018 Nov 28;7:97. doi: 10.1038/s41377-018-0098-6 (PMC6261969; doi:10.1038/s41377-018-0098-6)
Supplement: Supplementary file 1 — Supplementary Material [file 41377_2018_98_MOESM1_ESM.pdf]

Macroscale fluorescence imaging against autofluorescence under ambient light

Ruikang Zhang,[†] Raja Chouket,[†] Marie-Aude Plamont,[†] Zsolt Kelemen,[‡] Agathe Espagne,[†] Alison G. Tebo,[†] Arnaud Gautier,[†] Lionel Gissot,[‡] Jean-Denis Faure,[‡] Ludovic Jullien,[*,†] Vincent Croquette,[*,¶,§] and Thomas Le Saux[*,†]

†*PASTEUR, Département de Chimie, École normale supérieure, PSL University, Sorbonne Université, CNRS, 75005 Paris, France.*

‡*Institut Jean-Pierre Bourgin, INRA, AgroParisTech, CNRS, Saclay Plant Sciences (SPS), UniversiteParis-Saclay, Versailles, France.*

¶*Laboratoire de Physique Statistique, École normale supérieure, PSL Research University, Université Paris Diderot Sorbonne Paris-Cité, Sorbonne Université, CNRS, 75005 Paris, France.*

§*Institut de biologie de l'École normale supérieure (IBENS), École normale supérieure, CNRS, INSERM, PSL Research University, 75005 Paris, France.*

E-mail: Ludovic.Jullien@ens.fr; Vincent.Croquette@ens.fr; Thomas.Lesaux@ens.fr

Additional Methods

Materials

Reversibly photoswitchable fluorescent proteins The RSFPs used in this study are Dronpa-2[1,2] and Padron,[3] which belong to the Dronpa[4] family. Dronpa-2 contains only one mutation M159T and Padron contains eight mutations: T59M, V60A, N94I, P141L, G155S, V157G, M159Y and F190S.

Plasmids The plasmids for bacterial expression of Dronpa-2, Padron and EGFP, and the plants (*Camelina* and *Arabidopsis*) expression vector expressing p35S::Dronpa-2; pCVMV::DsRED construct have been previously described.[5]

Protein production and purification The plasmid expressing Dronpa-2 carrying an N-terminal hexahistidine tag was transformed in *E. coli* DH10B strain. Cells were grown in Terrific Broth (TB). Expression was induced by addition of isopropyl β-D-1-thiogalactopyranoside (IPTG) to a final concentration of 1 mM at OD(600 nm)=0.6. Cells were harvested after 16 h of expression and lysed by sonication in Lysis buffer (30/40 mM imidazole, 50 mM Tris/HCl at pH 7.5, 400 μM 1-(2-Aminoethyl) benzenesulfonyl fluoride hydrochloride, 5 mg/mL DNAse, 5 mM $MgCl_2$ and 1 mM dithiothreitol). Insoluble materials were removed by centrifugation and the soluble protein extract was batch-absorbed onto Ni-NTA agarose resin (Thermofisher). The protein loaded Ni-NTA column was washed with 20 column volumes of 50 mM TRIS/HCl pH 7.5, 20 mM imidazole, 150 mM NaCl. Bound protein was eluted in 50 mM TRIS/HCl pH 7.5, 500 mM imidazole, 150 mM NaCl. Protein fractions were dialyzed on cassette Slide-A-Lyzer Dialysis Cassettes (Thermofisher) against 50 mM TRIS/H_2SO_4 pH 8.0.

Preparation of the Dronpa-2 solutions at different concentrations The actual concentration of the purified Dronpa-2 stock solution was determined with a spectrophotometer (Agilent Technologies, Santa Clara, CA). Its UV-Vis absorption spectrum was recorded from 400 to 600 nm in a 55

μL quartz cuvette with 1.5 mm light path (Hellma Optics, Jena, Germany). The absorbance (0.16) at 480 nm yielded 29 μM concentration using $\varepsilon(480)$=37000 $M^{-1} \cdot cm^{-1}$ (evaluated after recording the absorbance at 447 nm of a denaturated Dronpa-2 solution in 1 M NaOH by using 44000 $M^{-1} \cdot cm^{-1}$ for the molar absorption coefficient of the deprotonated chromophore[6]). The Dronpa-2 stock solution was diluted with a BSA buffer (50 mM phosphate, 150 mM NaCl, 0.1 mM BSA, pH=7.4 measured with a Standard pH meter PHM210, equipped with a Radiometer Analytical PHC3359-8 combination pH electrode (Hach, Loveland, CO)) to generate a series of Dronpa-2 solutions at concentrations 20, 10, 5, 2, 1, 0.5, 0.2, 0.1, 0.05, and 0.02 nM.

Blot samples 1 μL aliquots of Dronpa-2 solutions in 0.1 mM BSA (Bovine Serum Albumin)–50 mM pH=7.4 PBS buffer at concentrations ranging from 20 to 0.02 nM were deposited on a nitrocellulose membrane (0.2 μm pore-size, 7× 8.5 cm; Bio-Rad, Hercules, CA, USA) by slightly pressing the micropipette tip onto the membrane. As the nitrocellulose membrane is hydrophilic, the Dronpa-2 solution permeates the membrane within a few seconds leading to the formation of a spot delimited by the tip area (diameter=0.4 mm).

Mixed bacterial culture Dronpa-2 and Padron were expressed in *E. coli* DH10B strain. Cells were grown into 2 mL of lysogeny broth (LB) at 37°C, 220 RPM for 1 h. Cells were platted at low density on LB agar plates, and plates were incubated overnight at 37°C. Dronpa-2 and Padron single colonies were then separately transfered to LB-ampicillin media and incubated at 37°C, 220 RPM for 4 h. The pre-cultures were diluted to reach OD(600 nm)=0.6, then mixed at the same concentrations, and were finally plated onto LB agar and incubated at 37°C.

Plant transformation and growth *Arabidopsis thaliana* and *Camelina sattiva*(cv Celine) were transformed with *Arabidopsis* floral-dip method and transgenic were selected as described previously.[7] *Arabidopsis* and *Camelina* seeds were sown respectively on sucrose-supplemented medium[8] or water-soaked paper and grown for 7 days in a growth chamber under cycles of 16 h light / 8 h dark at 22°C.

Optical design of the Speed OPIOM macroscope

Illumination system

The illumination system aims at increasing the distance between the last optical surface of our device and the sample without significant loss of light intensities delivered to the sample. The optical layout of the illumination system is shown in Fig.S1. Our design integrates one divergent doublet (ACN254-040-A, f = -40 mm, Thorlabs, NJ, US) and two convergent doublets (AC508-100-A, f = 100 mm, Thorlabs, NJ, US), which were chosen to minimize the spherical aberration at both 405 and 480 nm wavelengths and maximize the numerical aperture of the illumination system. The air spaces between the three elements (which define the size of the illuminated zone and the numerical aperture of the optical system) were optimized with the OSLO software (Lambda Research Corporation, Littleton, MA). Our final optical design allowed us to focus light emitted from the LEDs to a small area of 4×4 mm^2 at 120 mm away from the last optical surface, with powerful intensity (NA = 0.19) and excellent uniformity.

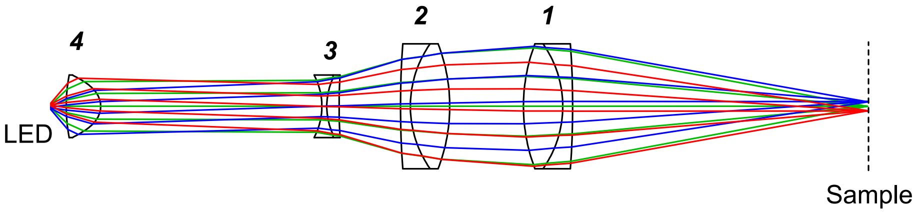

Figure S1: Optical layout of the illumination system. Optical elements denoted from ***1*** to ***4*** are given in Supplementary Table S2.

Imaging system

The macroscope aims at observing fluorescence emission from a small area within 4×4 mm^2 with both green (525 nm) and red (585 nm) channels. Before being imaged by the objective, the fluorescence emission is first collected by the beam-expanding system used for excitation, through which optical aberrations (especially the chromatic one) are introduced. Our objective consists of three lenses with different glass materials (BK7, SF11) to correct for chromatic aberration. The shape of each lens minimizing the spherical aberration at large NA and off-axis aberrations up to 2

mm from the optical axis was optimized using the OSLO software. Commercial lenses shapes and focal lengths at the closest to the optimized elements were chosen and the air space between each element was optimized to yield the final design (Fig. S2), which surpasses the imaging performances of singlet or achromatic doublet lenses (Fig. S3 and Fig. S4) for generating high quality images (Fig. S5).

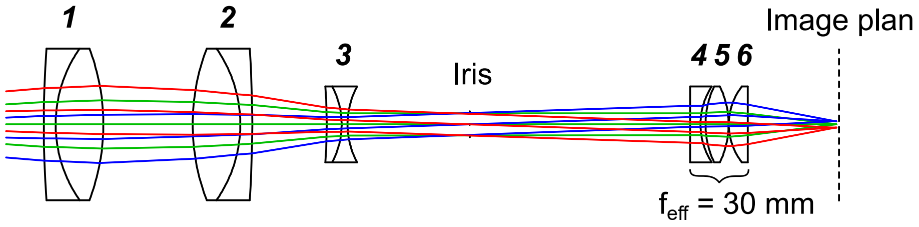

Figure S2: Optical layout of the imaging system. The final objective has an effective focal length of 30 mm, giving a magnification of 0.5× with a maximal usable aperture of F/4.0 (NA=0.125). The optical elements denoted from ***1*** to ***6*** are given in Supplementary Table S3.

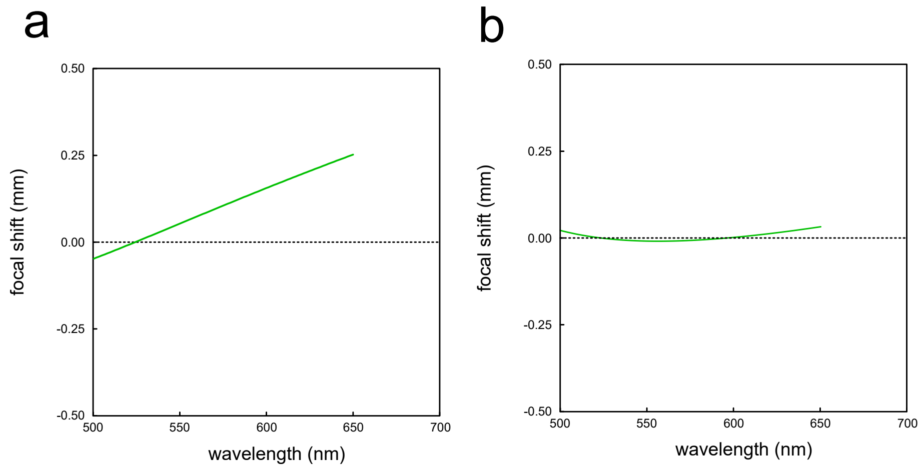

Figure S3: Simulated chromatic aberrations of the imaging system with an imaging objective composed of an achromatic doublet lens (f = 30 mm; **a**) or our designed lens system (**b**). Our optical design lowers by a factor ten the chromatic aberration.

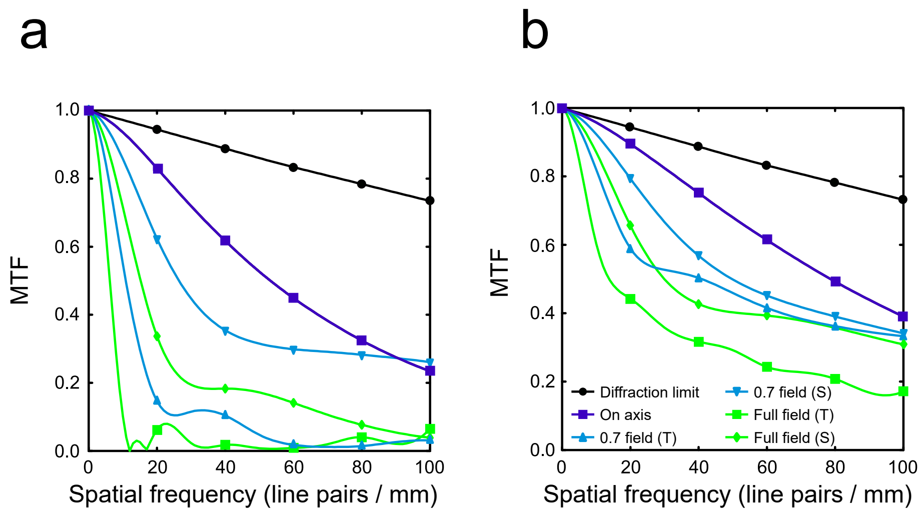

Figure S4: Modulation transfer function (MTF) in image space computed with an imaging objective composed of an achromatic doublet lens (f = 30 mm; **a**) or our designed lens system (**b**). Our optical design improves the overall resolution of the imaging system.

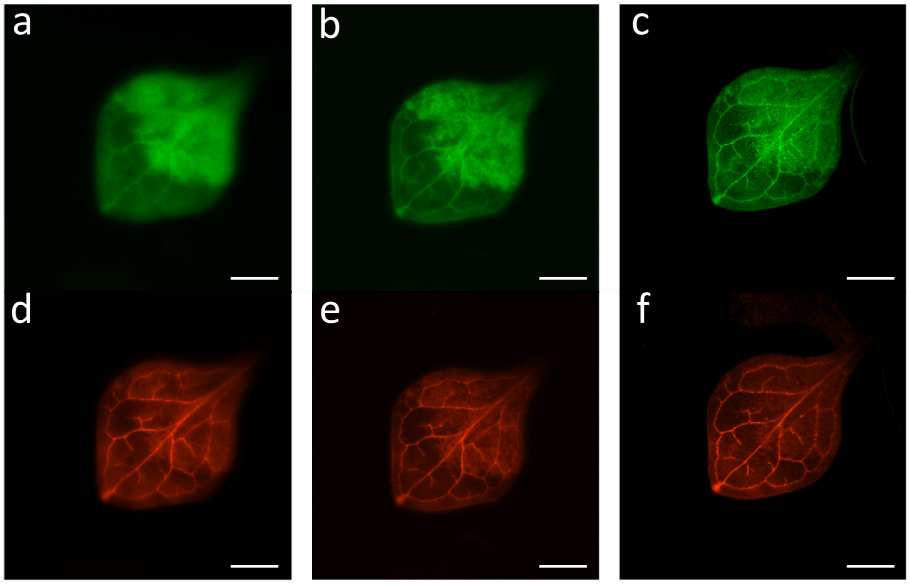

Figure S5: Quality of images of *Arabidopsis Thaliana* leaves observed in the red and green channels. **a,d**: Singlet lens; **b,e**: Achromatic lens (doublets); **c,f**: Designed lens system. Images taken with green filter ET525/36m and red filter ET585/20m, aperture F/4.0, focusing in the red channel. The system succeeds to improve the sharpness for both channels and corrected the defocus in the green channel. *Scale bars*: 1 mm.

Estimated cost of the Speed OPIOM macroscope

We currently estimate the cost of our imaging macroscope in the lower 10 k€ range as follows: camera 0.8 k€ , optomechanical parts /filters 7 k€, electronics (Teensy 3.5, LEDs and printed circuits boards) 0.2 k€, computer 1 k€.

Supplementary Figures

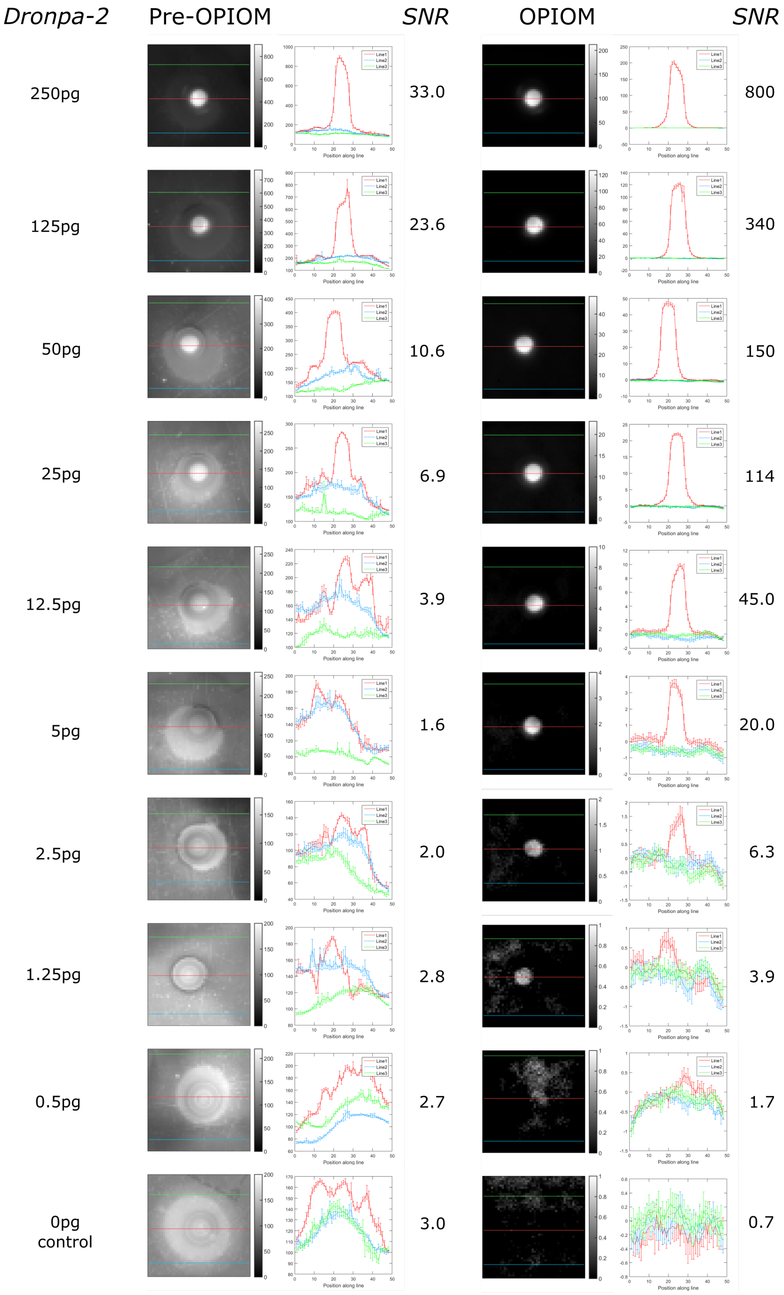

Figure S6: Dependence of the Pre-OPIOM and OPIOM images, of the signal profiles, and the associated signal-to-noise ratios of the nitrocellulose membrane on the Dronpa-2 amount (in pg) deposited on a 400 μm-diameter blot. In the signal profiles, the error bar measures the standard deviation over three independent measurements. The analyses have been performed along three lines: one crosses the middle of the blot (displayed in red) whereas the two others (shown in green and blue) are located out of it. The images have been recorded at resonance for Dronpa-2 (see also Table S1). One should notice that at low concentrations, the signal-to-noise analysis of the Pre-OPIOM images is no longer reliable since the signal observed at the blotted area is lower than the observed standard deviation of the membrane signal and much similar to the Pre-OPIOM signal observed for the control.

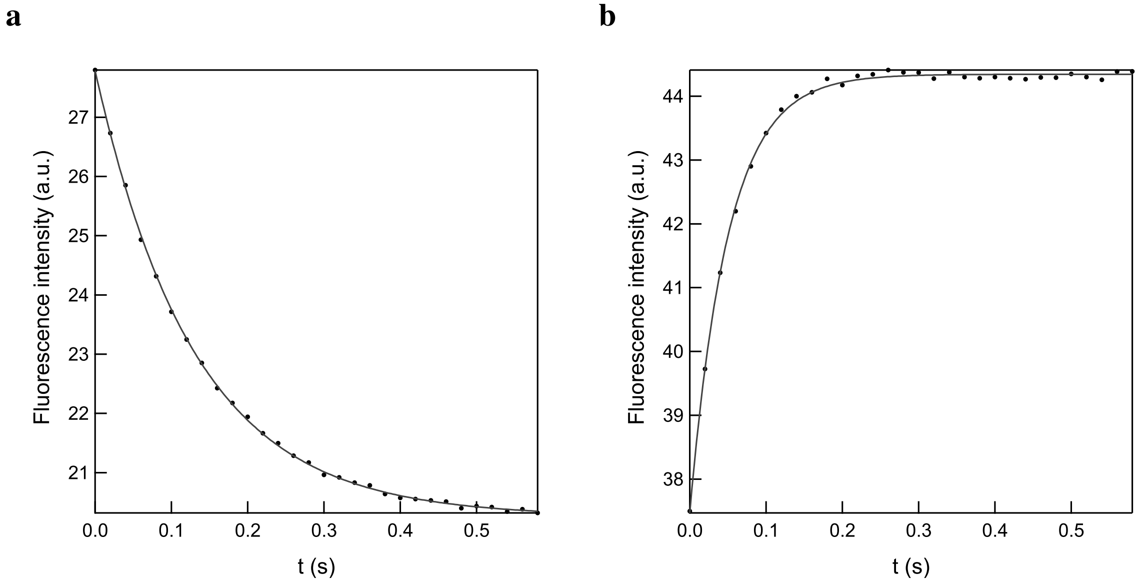

Figure S7: Photoisomerization kinetics of Dronpa-2. **a**: Evolution of the fluorescence emission of Dronpa-2 upon illumination at 480 nm (I_1^0 = 4×10^{-2} Ein.m^{-2}s^{-1}); **b**: Evolution of the fluorescence emission of Dronpa-2 upon illumination at both 480 and 405 nm (I_1^0 = 4×10^{-2} Ein.m^{-2}s^{-1} and I_2^0 = 1.9×10^{-2} Ein.m^{-2}s^{-1}).

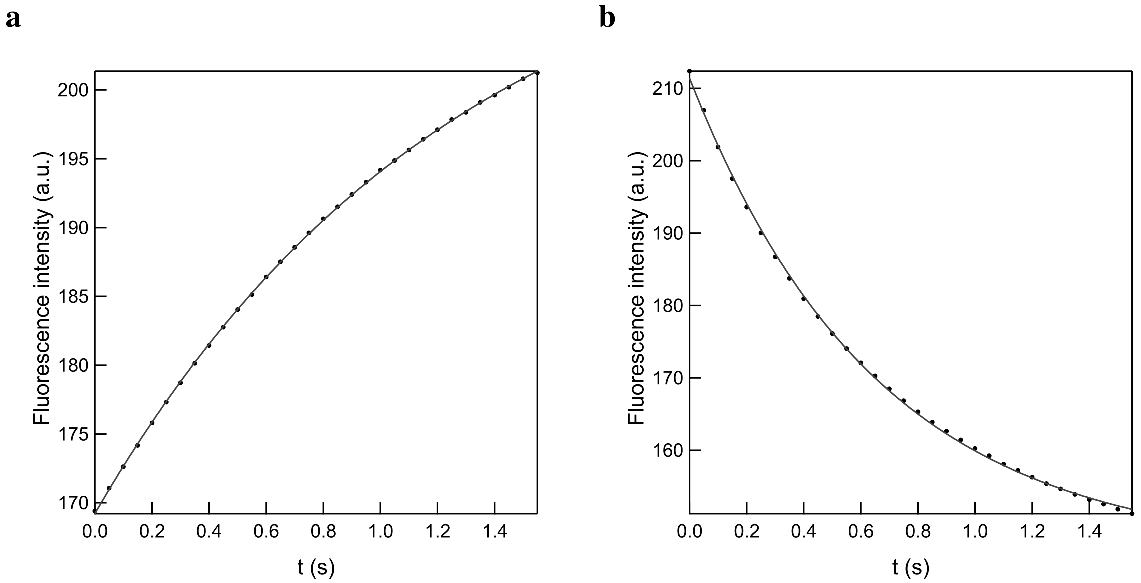

Figure S8: Photoisomerization kinetics of Padron. **a**: Evolution of the fluorescence emission of Padron upon illumination at 480 nm (I_1^0 = 1.75×10^{-2} Ein.m^{-2}s^{-1}); **b**: Evolution of the fluorescence emission of Padron upon illumination at both 480 and 405 nm (I_1^0 = 4.2×10^{-2} Ein.m^{-2}s^{-1} and I_2^0 = 6.5×10^{-3} Ein.m^{-2}s^{-1}).

Supplementary Table

Table S1: Acquisition parameters used for Speed OPIOM imaging in Figures 3,5,6 of the Main Text, where f_s refers to the sampling rate and f_m refers to the modulation frequency of the excitation lights.

Figure	Aperture	Periods	f_s (Hz)	f_m (Hz)	$\lambda_{exc,1}$ nm	I_1^0 Ein.m^{-2}.s^{-1}	$\lambda_{exc,2}$ nm	I_2^0 Ein.m^{-2}.s^{-1}
3a-j	F/4.0	8	50	2.5	480	4×10^{-2}	405	1.9×10^{-2}
5a,b,d-g	F/5.6	8	50	5	480	4×10^{-2}	405	1.9×10^{-2}
5c,h-j	F/5.6	2	4	0.02	480	4.2×10^{-2}	405	6.5×10^{-3}
6a-h	F/5.6	8	200	2.5	480	4×10^{-2}	405	1.9×10^{-2}

Table S2: Optical surfaces introduced in simulation of the illumination system.

Surface	Optical element	Radius (mm)	Thickness (mm)	Aperture Radius (mm)	Material
Object	LED	–	6.973/6.787[1]	0.5	air
1	(*4*)	69.999	14	12.7	B270
2		-8.818 (aspheric[2])	90	12.7	air
3	(*3*)	-27.05	2.00	12.7	N-BAF10
4		27.05	5	12.7	N-SF11
5		189.23	25.0	12.7	air
6	(*2*)	363.10	4	25.4	SF10
7		44.17	16.00	25.4	N-BAF10
8		-71.12	30.00	25.4	air
9	(*1*)	71.12	16.00	25.4	N-BAF10
10		-44.17	4.00	25.4	SF10
11		-363.1	120	25.4	air
Image		–	0	2.13	

[1]The radius were set at 6.973 and 6.787 mm for LED emitting light at 405 and 480 nm respectively.

[2]Aspheric coefficient for the $Z(r) = r^2/(R(1+\sqrt{1-(1+k)\frac{r^2}{R^2}})+A_4r^4+A_6r^6+A_8r^8+A_{10}r^{10}$ surface profile (sag) of the condenser: $R = 8.818$ $k = 0.9991715$, $A_4 = -8.6821674 \times 10^{-05}$, $A_6 = -6.3760123 \times 10^{-08}$, $A_8 = -2.4073084 \times 10^{-09}$ and $A_{10} = -1.7189021 \times 10^{-11}$, where r is the radial distance from the optical axis, R is the radius, k is the conic constant and A_4, A_6, A_8 and A_{10} are respectively the 4th, 6th, 8th and 10th order aspheric coefficients.

Table S3: Optical surfaces introduced in simulation of the imaging system.

Surface	Optical element	Radius (mm)	Thickness (mm)	Aperture Radius (mm)	Material
Object	Sample	–	120.00	2	air
1	(***1***)	363.10	4	25.4	SF10
2		44.17	16.00	25.4	N-BAF10
3		-71.12	30.00	25.4	air
4	(***2***)	71.12	16.00	25.4	N-BAF10
5		-44.17	4.00	25.4	SF10
6		-363.10	25.00	25.4	air
7	(***3***)	-189.23	5.00	12.7	N-SF11
8		-27.05	2.00	12.7	N-SF11
9		27.05	41.00	12.7	air
10	Iris	–	74.00	3.79	air
11	(***4***)	–	3.50	12.7	N-SF11
12		23.4	1.648	12.7	air
13	(***5***)	29.50	7.70	12.7	N-BK7
14		-29.50	0.1	12.7	air
15	(***6***)	20.60	6.4	12.7	N-BK7
16		–	29.767	12.7	air
Image		–	0	1.02	

References

(1) Ando, R.; Flors, C.; Mizuno, H.; Hofkens, J.; Miyawaki, A. Highlighted Generation of Fluorescence Signals Using Simultaneous Two-Color Irradiation on Dronpa Mutants. *Biophys. J.* **2007**, *92*, L97 – L99.

(2) Stiel, A. C.; Trowitzsch, S.; Weber, G.; Andresen, M.; Eggeling, C.; Hell, S. W.; Jakobs, S.; Wahl, M. C. 1.8 Å bright-state structure of the reversibly switchable fluorescent protein Dronpa guides the generation of fast switching variants. *Biochem. J.* **2007**, *402*, 35–42.

(3) Andresen, M.; Stiel, A. C.; Follig, J.; Wenzel, D.; Schoenle, A.; Egner, A.; Eggeling, C.; Hell, S. W.; Jakobs, S. Photoswitchable fluorescent proteins enable monochromatic multilabel imaging and dual color fluorescence nanoscopy. *Nat. Biotech.* **2008**, *26*, 1035–1040.

(4) Ando, R.; Mizuno, H.; Miyawaki, A. Regulated Fast Nucleocytoplasmic Shuttling Observed by Reversible Protein Highlighting. *Science* **2004**, *306*, 1370–1373.

(5) Quérard, J. et al. Resonant out-of-phase fluorescence microscopy and remote imaging overcome spectral limitations. *Nat. Comm.* **2017**, *8*, 969.

(6) Kredel, S.; Nienhaus, K.; Oswald, F.; Wolff, M.; Ivanchenko, S.; Cymer, F.; Jeromin, A.; Michel, F. J.; Spindler, K.-D.; Heilker, R.; Nienhaus, G. U.; Wiedenmann, J. Optimized and Far-Red-Emitting Variants of Fluorescent Protein eqFP611. *Chemistry & Biology* **2008**, *15*, 224 – 233.

(7) Morineau, C.; Bellec, Y.; Tellier, F.; Gissot, L.; Z. Kelemen, F. N.; Faure, J.-D. Selective gene dosage by CRISPR-Cas9 genome editing in hexaploid Camelina sativa. *Plant Biotechnol. J.* **2017**, *15*, 729–739.

(8) Estelle, M. A.; Somerville, C. Auxin-resistant mutants of Arabidopsis thaliana with an altered morphology. *Molecular and General Genetics MGG* **1987**, *206*, 200–206.
